# Patients with ROS1 rearrangement‐positive non‐small‐cell lung cancer benefit from pemetrexed‐based chemotherapy

**DOI:** 10.1002/cam4.809

**Published:** 2016-08-20

**Authors:** Zhengbo Song, Haiyan Su, Yiping Zhang

**Affiliations:** ^1^ Department of Medical OncologyZhejiang Cancer HospitalHangzhou310022China; ^2^ Key Laboratory Diagnosis and Treatment Technology on Thoracic OncologyZhejiang provinceHangzhou310022China; ^3^ Zhangzhou Municipal Hospital of Fujian ProvinceZhangzhou363000China

**Keywords:** Efficacy, non‐small‐cell lung cancer, pemetrexed, *ROS1*, thymidylate synthetase

## Abstract

*ROS1* gene‐rearrangement in non‐small‐cell lung cancer (NSCLC) patients has recently been identified as a driver gene and benefited from crizotinib treatment. However, no data are available for *ROS1*‐positive NSCLC about chemotherapeutic options and prognostic data. We investigated pemetrexed‐based treatment efficacy in *ROS1* translocation NSCLC patients and determined the expression of thymidylate synthetase (TS) to provide a rationale for the efficacy results. We determined the *ROS1* status of 1750 patients with lung adenocarcinoma. Patients' clinical and therapeutic profiles were assessed. In positive cases, thymidylate synthetase (*TS*) mRNA level was performed by RT‐PCR. For comparison, we evaluated the TS mRNA status and pemetrexed‐based treatment efficacy from 170 NSCLC patients with anaplastic lymphoma kinase (*ALK*) translocation (*n* = 46), *EGFR* mutation (*n* = 50), *KRAS* mutation (*n* = 32), and wild‐type of *EGFR/ALK/ROS1/KRAS* (*n* = 42). Thirty‐four ROS1 translocation patients were identified at two institutions. Among the 34 patients, 12 with advanced stage or recurrence were treated with pemetrexed‐based first‐line chemotherapy. The median progression‐free survivals of pemetrexed‐based first‐line chemotherapy in *ROS1* translocation, *ALK* translocation, *EGFR* mutation, *KRAS* mutation, and *EGFR/ALK/ROS1/KRAS* wild‐type patients were 6.8, 6.7, 5.2, 4.2, and 4.5 months, respectively (*P* = 0.003). The *TS* mRNA level was lower in patients with *ROS1*‐positive than *ROS1*‐negative patients (264 ± 469 × 10^−4^ vs. 469 ± 615 × 10^−4^, *P* = 0.03), but similar with *ALK*‐positive patients (264 ± 469 × 10^−4^ vs. 317 ± 524 × 10^−4^, *P* = 0.64). Patients diagnosed with *ROS1* translocation lung adenocarcinoma may benefit from pemetrexed‐based chemotherapy. TS mRNA level enables the selection of therapeutic options for *ROS1* translocation patients.

## Introduction

Chromosomal rearrangements of the gene encoding *ROS1* proto‐oncogene receptor tyrosine kinase (*ROS1*) define a distinct molecular subset of non‐small‐cell lung cancer (NSCLC)^1^. *ROS1* rearrangements occur in approximately 1% to 2% of patients with NSCLC^2^, ^3^. *ROS1* fusion has been identified as a driver gene and fusion partners including *CD74, SLC34A2, TPM3, SDC4, EZR, LRIG3, KDELR2,* and *CCDC6* have been identified^4^. Due to highly similar tyrosine kinase domains, preclinical and clinical data suggest that *ROS1* can be targeted by *ALK* inhibitors^5^. Several studies with limited number of patients demonstrated that crizotinib, an *ALK* inhibitor, was effective against NSCLC in patients harboring *ROS1* translocation^6^, ^7^. In China, crizotinib is not commonly used for *ROS1* translocation NSCLC patients due to lack of efficacy and toxicity data. Chemotherapeutic agents are the standard of care for *ROS1* translocation NSCLC patients currently. For patients with *ROS1* translocation, platinum‐based chemotherapy is recommended currently in clinical practice.

The efficacy of pemetrexed‐based treatment is superior to that of other regimens in *ALK* translocation patients^8^, ^9^, ^10^, ^11^. In addition, low TS level was significantly associated with better clinical efficacy in nonsquamous NSCLC patients who were treated with pemetrexed‐based chemotherapy^8^. Recent studies demonstrated that *TS* mRNA levels in *ALK* translocation tumor specimens were significantly lower compared with *ALK* translocation tissue, which may partially explain the clinical outcome of pemetrexed‐based chemotherapy in this population 8, 11. However, for lower frequency of *ROS1* translocation than *ALK* translocation NSCLC, the *TS* mRNA level in *ROS1* translocation samples is not well investigated 12. No clinical efficacy data of pemetrexed‐based chemotherapy efficacy exists currently.

In this study, we have carried out an analysis of prevalence and clinical efficacy of pemetrexed‐based chemotherapy of *ROS1* translocation NSCLC patients, and further investigated *TS* mRNA levels in *ROS1*‐positive and *ROS1*‐negative patients.

## Materials and Methods

### Study populations

Consecutive patients who had been diagnosed with NSCLC between January 2010 and December 2014, at Zhejiang Cancer Hospital and Zhangzhou Municipal Hospital were screened for *ROS1* gene. For comparison, 170 patients identified as *EGFR* mutation, *ALK* translocation, *KRAS* mutation, and *EGFR/ALK/ROS1/KRAS* wild‐type during the same period were collected. Tumor histology was classified according to the World Health Organization criteria (2004 version)^13^. Lung cancer staging was performed in all patients according to the 7th TNM classification. This study was approved by the Institutional Review Board of the two institutions.

### Genetic analysis and measurement of TS mRNA

The *EGFR/ALK/ROS1/KRAS* detection Kit (Amoy Diagnostics, Xiamen, China) is based on the reverse‐transcriptase polymerase chain reaction (RT‐PCR) technology. All positive samples were validated with Sanger sequencing. All experiments were performed following the user manual. Details of the method have been described in previous report^14^. RNA was extracted for thymidylate synthase (*TS*) mRNA level from unstained sections according to standard protocols. Relative quantification for the TS genes was done using a quantitative RT‐PCR detection method (LightCycler 2.0; Roche Applied Science, Penzberg, Germany) and an internal reference gene (*β*‐actin) as a control. The details of the operation were carried out as previously described^11^.

### Statistical analysis

Fisher's exact test and Wilcoxon rank‐sum test were used to assess the baseline characteristics of the different groups. Progression‐free survival (PFS) was estimated by the Kaplan–Meier method, and the log‐rank test was used to compare the differences between the groups. The data analysis was performed using Statistics 17.0 (SPSS Inc, Chicago, IL, USA). The last follow‐up time was March 1, 2015.

## Results

### Clinicopathologic characteristics of ROS1‐positive patients

Thirty‐four patients including 16 males and 18 females with lung adenocarcinoma were identified as *ROS1* translocation with a frequency of 1.9% (34/1750). The clinicopathologic characteristics of these patients are listed in Table 1. Four patients have smoking history. According to the pathologic and clinical stages, 15 patients were diagnosed with stage IIIB/IV, and 19 with stage I to IIIA at diagnosis.

**Table 1:** Clinicopathologic features comparison among patients harboring different genes

	*ROS1* (*n* = 34)	*ALK* (*n* = 46)	*KRAS* (*n* = 32)	*EGFR* (*n* = 50)	*wild‐type* (*n* = 42)	*P* (*ROS1* vs*. ALK*)	*P* (*ROS1* vs*. KRAS*)	*P* (*ROS1* vs*. EGFR*)	*P* (*ROS1* vs*. wild‐type*)
Gender						0.90	0.02	0.32	0.28
Male	16	21	24	29	25				
Female	18	25	8	21	17				
Age at diagnosis						0.89	0.13	0.67	0.85
<60	19	28	13	32	24				
≥60	13	18	19	18	18				
Smoking history						0.31	<0.001	0.007	0.0044
Yes	5	11	22	21	19				
No	29	35	10	29	23				
Histology						0.61	0.44	0.24	0.89
Adenocarcinoma	34	44	30	46	41				
Nonadenocarcinoma	0	2	2	4	1				
Stage at diagnosis						0.80	0.46	0.99	0.76
I‐IIIA	19	27	15	28	22				
IIIB/IV	15	19	17	22	20				
Pemetrexed‐based chemotherapy						0.41	0.42	0.48	0.57
First‐line	12	24	19	28	23				
Second or third‐line	6	7	4	9	8				

### Clinicopathologic comparison

Totally, 46 patients with *ALK* translocation, 32 with *KRAS* mutation, 50 with *EGFR* mutation, and 42 with *EGFR/ALK/ROS1/KRAS* wild‐type were selected in this study concurrently with *ROS1*‐positive patients. Clinicopathologic comparison of patients with different gene types is presented in Table 1. There was a difference of smoking history between *ROS1*‐positive and *ROS1*‐negative patients. No difference was found in gender, age, stage, and histology among different groups.

### Treatment efficacy and comparison

**Figure 1:**
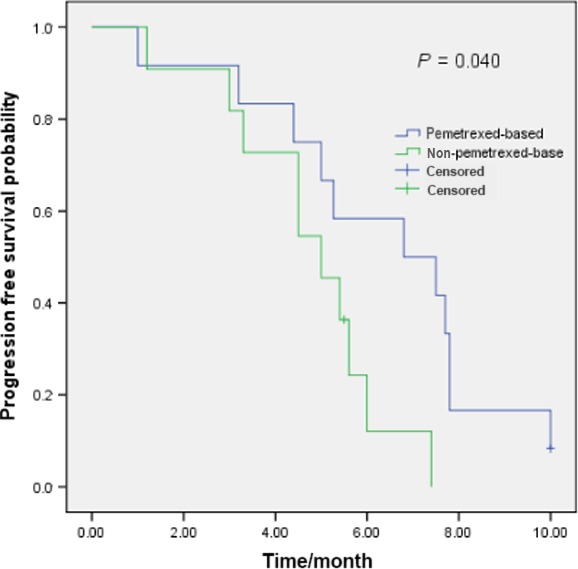
Pemetrexed‐based (*n* = 12) and non‐pemetrexed‐based(*n* = 11) first‐line chemotherapy efficacy comparison in ROS1‐positive NSCLC patients.

**Figure 2:**
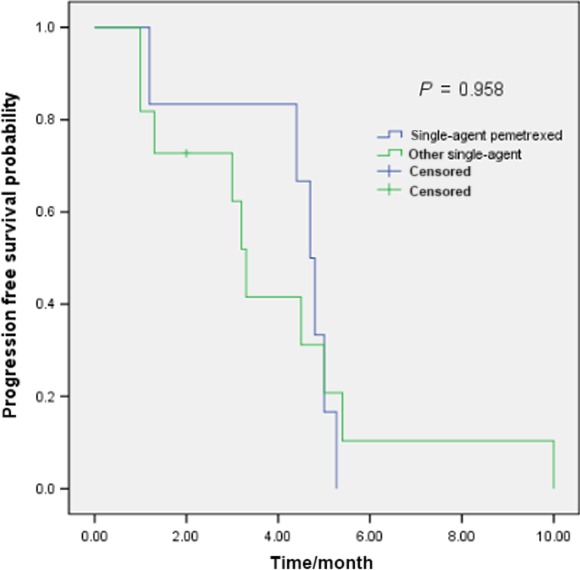
Single‐agent pemetrexed (*n* = 6) and other single‐agent (*n* = 13) chemotherapy efficacy comparison in ROS1‐positive NSCLC patients.

Totally, 23 patients with *ROS1* translocation received first‐line palliative chemotherapy (15 with stage IIIB/IV at diagnosis and eight with recurrence). Twelve *ROS1* translocation patients received platinum/pemetrexed first‐line treatment, and the other 11 patients received platinum‐based double regimens (seven with gemcitabine and four with docetaxel). Six patients received single‐agent pemetrexed as second or third‐line treatment and 13 cases received another single‐agent. No maintenance therapy was used after first‐line treatment in all of patients. The median PFS with first‐line platinum/pemetrexed was 6.8 months, while the median PFS with other platinum‐based double regimens was 5.0 months (*P* = 0.040)(Fig. 1). The median PFS of patients receiving single‐agent pemetrexed was 4.7 months. In contrast, the median PFS with other single‐agent was 3.3 months (*P* = 0.958)(Fig. 2).

**Figure 3:**
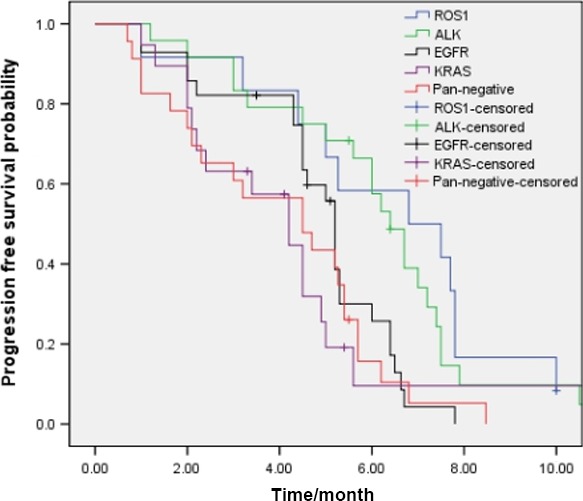
Pemetrexed‐based first‐line chemotherapy efficacy comparison in NSCLC patients with ROS1‐positive(*n* = 12), ALK‐positive (*n* = 24), KRAS‐positive(*n* = 19), EGFR‐positive (*n* = 28), and pan‐negative patients (*n* = 23) (*P* = 0.003).

Totally, for the 170 patients selected for comparison, there were 27, 22, 34, and 27 patients with *ALK* translocation, *KRAS* mutation, *EGFR* mutation, and *EGFR/ALK/ROS1/KRAS* wild‐type who received first‐line platinum/pemetrexed treatment. The median PFS of first‐line platinum/pemetrexed chemotherapy for all the patients was 5.2 months. The PFS was 6.8, 6.7, 5.2, 4.2, and 4.5 months in ROS1 translocation, *ALK* translocation, *EGFR* mutation, *KRAS* mutation, and *EGFR/ALK/ROS1/KRAS* wild‐type patients, respectively (*P* = 0.003)(Fig. 3).

A difference in PFS of the first‐line platinum/pemetrexed chemotherapy was apparent between *ALK/ROS1‐positive* and *ALK/ROS1‐*negative patients (6.7 vs. 4.6 months, P<0.001), while no difference was seen between *ALK* translocation and *ROS1* translocation patients (6.8 vs. 6.7 months, *P* = 0.498).

**Figure 4:**
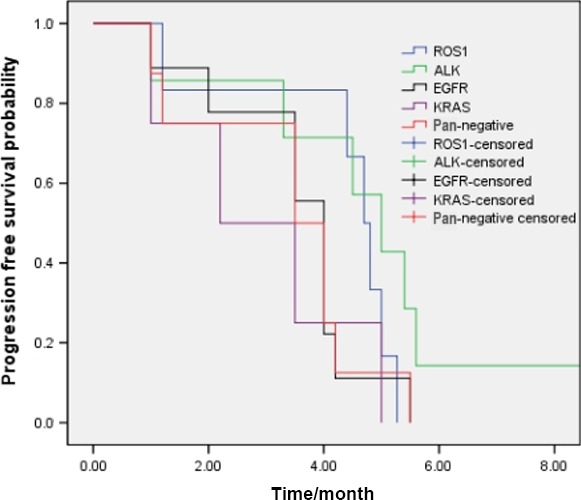
Single‐agent pemetrexed chemotherapy efficacy comparison in NSCLC patients with ROS1‐positive(*n* = 6), ALK‐positive (*n* = 7), KRAS‐positive(*n* = 4), EGFR‐positive (*n* = 9), and pan‐negative patients (*n* = 8)(*P* = 0.218).

For patients with single‐agent pemetrexed treatment, no efficacy difference was found among the *ROS1* translocation, *ALK* translocation, *EGFR* mutation, *KRAS* mutation, and *EGFR/ALK/ROS1/KRAS* wild‐type patients. (*P* = 0.218)(Fig. 4).

### Thymidylate synthase (TS) mRNA level comparison

**Figure 5:**
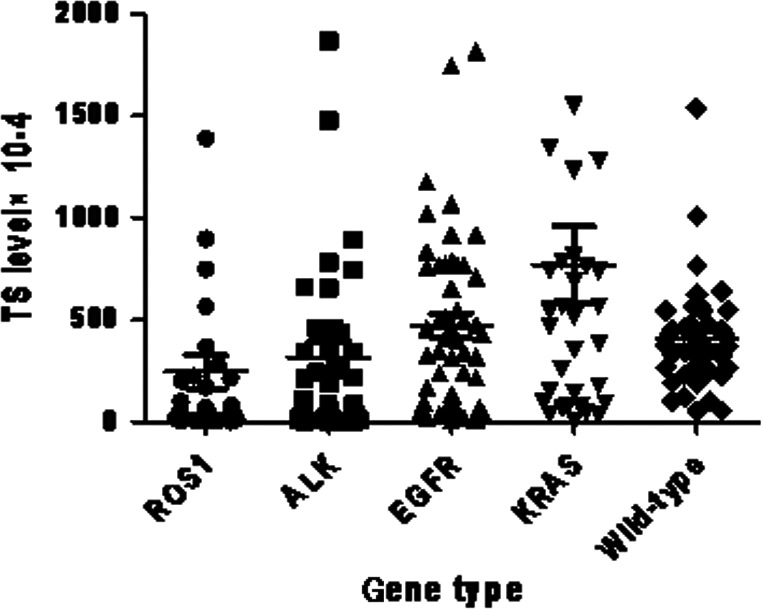
Thymidylate synthase (TS) mRNA levels in NSCLC patients with different driver genes.

Expression levels of TS mRNA are showed in Figure 5. The *TS* mRNA levels in *ROS1* translocation, *ALK* translocation, *EGFR* mutations, *KRAS* mutations, and *EGFR/ALK/ROS1/KRAS* wild‐type patients were 264 ± 469 × 10^−4^, 317 ± 524 × 10^−4^, 470 ± 422 × 10^−4^, 770 ± 1085 × 10^−4^, and 407 ± 251 × 10^−4^, respectively.

*TS* mRNA levels in *ROS1* translocation patients were significantly lower than in patients with *KRAS* mutation (*P* = 0.015) and *EGFR* mutation (*P* = 0.043), respectively. There was a tendency to lower TS levels in *ROS1* translocation than in *EGFR/ALK/ROS1/KRAS* wild‐type patients (*P* = 0.11). No difference of *TS* mRNA level was found between *ROS1* translocation and *ALK* translocation group (*P* = 0.64)(Fig. 5).

## Discussion

We have carried out the study to determine the efficacy of pemetrexed‐based chemotherapy efficacy and the *TS* mRNA levels in *ROS1* translocation lung adenocarcinoma. Our results suggest that *ROS1* translocation patients manifest longer PFS with pemetrexed‐based chemotherapy and lower *TS* mRNA levels compared with ROS1 translocation patients.

To date, no more than one hundred *ROS1* translocation patients regardless of retrospective or prospective study treated with crizotinib have been reported. In one study by Shaw, et al., 50 *ROS1* translocation patients showed good efficacy with crizotinib treatment with a median PFS of 19.2 months^6^. Together with EUROS1 study^7^, ROS1 rearrangement defines another molecular subtype of NSCLC against which crizotinib is highly active. Due to the relative rarity of *ROS1* translocation NSCLC, a randomized trial comparing crizotinib with chemotherapy is difficult. Pemetrexed is a multi‐targeted antifolate agent that disrupts the nucleotide syntheses of pyrimidines and purines, resulting in potent antitumor activity 15. Pemetrexed‐based chemotherapy is recommended as first‐line, maintenance and second‐line standard treatment regimens in nonsquamous NSCLC patients 16, 17, 18. Several randomized trials have shown that TS‐negative NSCLC patients benefited from pemetrexed‐based chemotherapy more than TS‐positive patients 19, 20. TS expression was identified as a potential predictive marker of efficacy in pemetrexed‐based regimens of NSCLC treatment.

The efficacy of pemetrexed‐based regimens in *ALK* translocation NSCLC patients has been identified in previous prospective and retrospective studies 8, 21, 22. *ALK* translocation patients treated with pemetrexed single‐agent showed a longer PFS than docetaxel in PROFILE 1007 study (4.2 months vs. 2.6 months)^21^. Similarly, the median PFS reached 7.0 months in *ALK* translocation patients treated with pemetrexed‐based first‐line chemotherapy in PROFILE 1014 study 22, which was longer than in previous reports of its efficacy 16, 17, 18. Several preclinical and clinical studies demonstrated that *TS* mRNA levels were significantly lower in patients harboring *ALK* translocation than in patients without *ALK* translocation, which may partially explain the outcome in patients amenable to pemetrexed‐based chemotherapy 8, 11. However, due to the rarity of *ROS1* translocation patients, few data are available for the chemotherapeutic efficacy of pemetrexed‐based therapy except for two case reports 12, 23, 24 and the *TS* mRNA levels are unclear currently.

Twelve patients of *ROS1* translocation were treated with pemetrexed‐based first‐line chemotherapy in our study. The outcome demonstrated that the PFS of this regimen was similar to that of *ALK* translocation patients, but longer than in patients without *ALK/ROS1* translocation status. Moreover, we detected the TS mRNA levels in patients with different genes and the results showed that *TS* mRNA levels were lower in *ROS1*‐positive than in *ROS1*‐negative patients, which partly explains the efficacy of pemetrexed‐based chemotherapy in this subset.

One of the major limitations of our study is related to the small sample with *ROS1* translocation patients. A second major limitation is that treatment regimens varied and not all of the patients were treated with pemetrexed‐based chemotherapy. Third, the retrospective nature of the study may be associated with sampling bias. However, due to lack of study to detect the clinical efficacy and *TS* mRNA levels in *ROS1* translocation patients, our study assumes clinical significance for clinical practice.

In summary, we have shown that *ROS1* translocation patients may benefit from pemetrexed‐based chemotherapy and *TS* mRNA levels are lower in this subset. For *ROS1* translocation NSCLC patients, a prospective study with large number of patients is needed to establish the clinical efficacy of different chemotherapy regimens.

## Conflicts of interest

None declared.
